# Difluoromethylornithine, a Decarboxylase 1 Inhibitor, Suppresses Hepatitis B Virus Replication by Reducing HBc Protein Levels

**DOI:** 10.3389/fcimb.2020.00158

**Published:** 2020-04-16

**Authors:** Binli Mao, Zhuo Wang, Sidie Pi, Quanxin Long, Ke Chen, Jing Cui, Ailong Huang, Yuan Hu

**Affiliations:** Key Laboratory of Molecular Biology on Infectious Diseases, Ministry of Education, Institute for Viral Hepatitis, Department of Infectious Diseases, Second Affiliated Hospital, Chongqing Medical University, Chongqing, China

**Keywords:** hepatitis B virus, DFMO, ODC1, polyamines, HBc

## Abstract

Current treatments of hepatitis B virus (HBV) are limited to Interferon-alpha or the nucleos(t)ide analogs antiviral therapies, and it is crucial to develop and define new antiviral drugs to cure HBV. In this study, we explored the anti-HBV effect of difluoromethylornithine (DFMO), an irreversibly inhibitor of decarboxylase 1(ODC1) on HBV replication. Firstly, we found that polyamines contributed to HBV DNA replication via increasing levels of the HBV core protein (HBc) and capsids. In contrast, depletion of polyamines either by silencing the expression of ODC1 or DFMO treatment, resulted in decreasing viral DNA replication and levels of HBc protein and capsids. Furthermore, we found that DFMO decreased the stability of the HBc protein without affecting mRNA transcription and protein translation. Taken together, our findings demonstrate that DFMO inhibits HBV replication by reducing HBc stability and this may provide a new approach for HBV therapeutics.

## Introduction

Despite employing an effective vaccine against Hepatitis B virus (HBV) infection, HBV remains a major serious health problem worldwide (Lampertico et al., 2017). There are about 257 million people chronically infected worldwide and over 887,000 death every year according to a WHO report (Revill et al., 2019). Chronic hepatitis B infection (CHB) often causes cirrhosis and liver cancer (Schweitzer et al., 2015). Currently, there are two approved antiviral treatments for CHB, including interferon-alpha and nucleotide analogs (NAs) (Block et al., 2015). However, due to the side effects of interferon-alpha or drug resistance for NAs (Zoulim and Locarnini, 2009), the current therapeutic efficacy is limited. Therefore, developing new drugs that directly target either virus or host factors for an efficient CHB treatment is viral and urgent (Mitra et al., 2018).

HBV is a small enveloped virus that encodes four overlapping open-reading frames (ORFs) including the HBV polymerase, the HBV core protein (HBc), envelope proteins, and the HBV X protein (HBx) (Seeger and Mason, 2015). Increasing evidence has indicated that HBc displays multiple complex functions during HBV replication (Diab et al., 2018), including capsid formation (Zlotnick et al., 2015) and epigenetic regulation of the cccDNA minichromosomal (Pollicino et al., 2006). Thus, HBc has been considered to be an attractive and promising target for anti-HBV therapy (Block et al., 2015; Durantel and Zoulim, 2016), and drugs targeting HBc are currently under development (Stray and Zlotnick, 2006; Yang et al., 2016; Ko et al., 2019).

**Figure 1:**
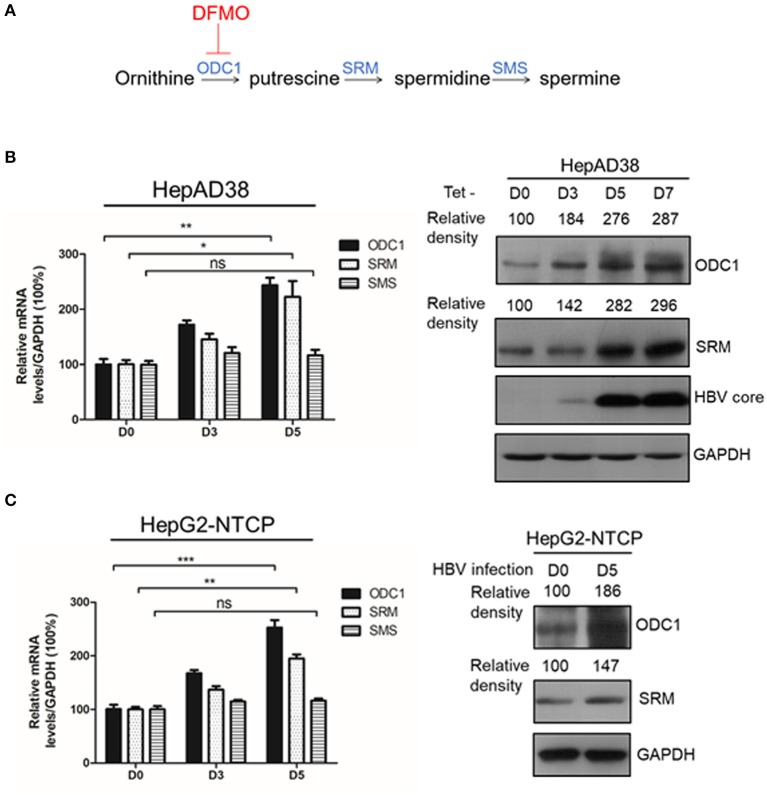
ODC1 and SRM in polyamine metabolism pathway are upregulated in HBV replicating hepatoma cells. **(A)** Schematic representation of polyamine metabolic pathway including enzymes and inhibitors. As shown in the image, DFMO could block ODC1 activity, which converts ornithine into the putrescine. Putrescine is converted into spermidine via spermidine synthase (SRM), the spermidine is then converted into spermine via spermine synthase (SMS). **(B)** Real-time RT-PCR (left panel) and Western blotting (right panel) analysis of ODC1, SRM and SMS expression levels in the HepAD38 cells after removing tetracycline for different time points. **(C)** Real-time RT-PCR (left panel) and Western blotting (right panel) analysis of ODC1, SRM and SMS expression levels in the HepG2-NTCP infected with HBV particles with different time points. The levels of ODC1 and SRM proteins were normalized to the levels of GAPDH and analyzed by the Image J software. Statistical significance was determined by one-way ANOVA with the Tukey *post-hoc* test (*p < 0.05, **p < 0.01, ***p < 0.001; ns, not significant). Data have been represented as the mean ± SD of three independent experiments.

Polyamines (including putrescine, spermidine and spermine) are small and positively charged molecules, which are involved in several cellular processes in mammalian cells, such as gene transcription, mRNA translation, cell growth and apoptosis (Igarashi and Kashiwagi, 2015; Miller-Fleming et al., 2015). Polyamines are implicated in more aspects of the replication cycle of several viruses, such as the herpes simplex viruses (HSV), where polyamines facilitate viral DNA packaging (Gibson and Roizman, 1971) or chikungunya virus (CHIKV) and Zika virus (ZIKV), where polyamines are necessary for translation of the viral mRNAs (Mounce et al., 2016b). In addition, Ornithine decarboxylase (ODC1), spermidine synthase (SRM) and spermine synthase (SMS) are rate-limiting enzymes in intracellular polyamine biosynthetic pathway (Raul, 2007; Figure 1A). As an irreversibly inhibitor of ODC1, difluoromethylornithine (DFMO) has been recently demonstrated to inhibit replication of diverse viruses such as Human Cytomegalovirus (HCMV) (Gibson et al., 1984) by reducing the levels of polyamines. DFMO can also inhibit replication of several RNA virus, such as the Dengue virus (DENV), the ZIKV, the CHIKV (Mounce et al., 2016a). These findings have strengthened the importance of the polyamines toward viral replication and highlighted the potential function of DFMO as a promising broad-spectrum antiviral drug. However, whether polyamines are involved in HBV life cycle or DFMO can inhibit HBV replication are remain unclear. In this study, we revealed that polyamines were required for HBV replication, and DFMO could restrict the viral DNA replication via reducing the HBc protein levels. These results highlight the potential role of DFMO as a promising therapeutic target for anti-HBV treatment.

## Materials and Methods

### Cell Culture and Transfection

HepAD38, HepG2, HepG2-NTCP and HepG2.2.15 cells were cultured in the Dulbecco's modified Eagle's medium (DMEM) supplemented with 10% fetal bovine serum (Biological Industries, Israel),100 U/mL penicillin (Gibco, Life Technologies, Carlsbad, CA, USA) and 100 g/mL streptomycin (Gibco, Life Technologies, Carlsbad, CA,USA). To maintain the stably transfected HBV genome, HepG2.2.15 cells were grown with 200 ug/mL G418. As for the HepAD38 cells, 1 μg/mL tetracycline was added to suppress HBV transcription.

The expression vector for 3xFlag-HBc was cloned with a N-terminal 3xFlag-tag in pEZ-M12 vector by Genecopoeia Company. The expression vectors for 3xFlag-HBx and 3xFlag-HBs are plasmids expressing the HBx and HBV surface antigen (HBs), respectively. Small interfering RNAs (siRNAs) were purchased from Shanghai Jima Company and the siRNA sequences targeting human ODC1, SRM, elF5A1 and elF5A2 have been showed in Supplement Table 1. Lipofectamine 3000 (Invitrogen, Carlsbad, CA, USA) was used for the transfection of plasmids or siRNAs according to the manufacturer's instructions.

### Chemical Reagents

DFMO was purchased from selleckchem company. Exogenous polyamines, spermidine and spermine were purchased from Sigma company. Cycloheximide (CHX) and carbobenzoxy-Leu-Leu-leucinal (MG132) were purchased from AbMole. All drugs were stored at −20°C until further use.

### RNA Purification and Real-Time RT-PCR

For RNA purification, cells were washed with PBS and total RNA was extracted by TriZol (Life Technologies, Carlsbad, CA, USA) according to the manufacturer's instructions. Purified RNA was transcribed into cDNA with Primescript RT reagent Kit with gDNA Eraser (Takara,Tokyo, Japan). Real-time RT-PCR was performed to determine the levels of target gene. Expression levels of GAPDH mRNA were used as an internal control, and the 2^−ΔΔ*Ct*^ method was used for the final evaluation. Primers have been shown in Supplement Table 1.

### Western Blotting

The methods for protein measurement in cell lysates and Western blotting were performed as described previously (Chen et al., 2018). The antibodies for immunoblots used in this study are follows: anti-HBc (B0586, Dako, Denmark), anti-flag (MA-1-91878, Thermo, USA), anti-ODC1(sc-398116, Santa Cruz, USA), anti-SRM (bs-17653R, Bioss, China), anti-elF5A (ET1610-49, Hangzhou Hua An Biotechnology, China), anti-HBs (NB100-62652, Novus, USA), anti-GAPDH (100242-MM05, Sino Biological, China). Quantifications of the immunoblot band intensities were analyzed by the Image J software.

### Enzyme-Linked Immunosorbent Assay (ELISA)

Hepatitis B surface antigen (HBsAg) in cell supernatant was detected using an ELISA assay kit (KHB, Shang Hai, China) according to the manufacturer's protocol.

### Virus Production and HBV Infection

For production of the HBV virions, supernatants of HepAD38 cells were filtered, precipitated with 10% PEG8000, and centrifuged as described previously (Chen et al., 2018). For HBV infection, the HepG2-NTCP cells were infected with HBV viral particles at 1,000 genome equivalents (GE) per cell in the presence of PEG8000. After removing virus from the infected cells, they were maintained in the Williams' E media before harvest.

### Extraction and Quantitative Analysis of HBV DNA by Southern Blotting and Real-Time PCR

The method for the extraction and detection of intracellular HBV core-associated DNA was conducted as described previously (Chen et al., 2018). Briefly, the intracellular HBV core-associated DNA was extracted through a sucrose density gradient and purified by phenol/chloroform, then the extracted viral DNA was electrophoresed on 1.0% agarose gels and transferred into nylon membranes (Roche, Basel, Switzerland). After immobilization on the membranes, the viral DNA was detected by using the DIG high prime DNA labeling and detection starter kit (Roche Diagnostics). For the assessment of the HBV core-associated DNA levels by real-time PCR was conducted as previously described (Hu et al., 2018).

### Native Gel Analysis of HBV Capsids

The method for the detection HBV core particles was conducted as described previously (Hu et al., 2018). Briefly, cell lysates were loaded on native 1% agarose gels, and the viral particles transferred onto a nitrocellulose (NC) membrane were probed with an anti-HBV core antibody (B0586, Dako, Denmark).

### Drug Viability Assay

The cytotoxic effects of drugs employed in this research on human hepatoma cells were detected by using the Celltiter 96 aqueous non-radioactive cell proliferation assay (Promega, Madison, WI, USA). For that, cells were seeded into 96-well plates and maintained with drugs for 3 days, followed by measuring the absorbance at 490 nm according to the manufacturer's instruction.

### Statistical Analysis

Statistics evaluations were performed using the GraphPad Prism8.0 (GraphPad Software, San Diego, CA, USA). Unpaired *t*-test or ANOVA by one way with Tukey *post-hoc* test was used to determine significant differences. Differences were considered as statistically significant as *p* < 0.05.

## Results

### ODC1 and SRM in Polyamine Metabolism Pathway Are Up-Regulated in HBV Replicating Hepatoma Cells

ODC1, SRM and SMS are rate-limiting enzymes involved in intracellular polyamine biosynthetic pathway (Figure 1A), therefore, we initially investigated the expression of these three enzymes in the presence of HBV. In HBV-stable expressing cells HepAD38, the HBV replication was inducted by removing the tetracycline. The expression of ODC1 and SRM, but not for SMS, was gradually increased both at mRNA and the protein levels with HBc expression (Figure 1B). Furthermore, we examined the expression of ODC1, SRM and SMS in HepG2-NTCP cells that support HBV infection. We also found that the expression of ODC1 and SRM increased both at mRNA and the protein levels by an HBV infection (Figure 1C). These results suggested that ODC1 and SRM that regulate the levels of cellular polyamines were upregulated in HBV replication and infection cell models.

### Silencing the Expression of ODC1 and SRM Decrease Levels of the HBV Core-Associated DNA and the HBc Protein

**Figure 2:**
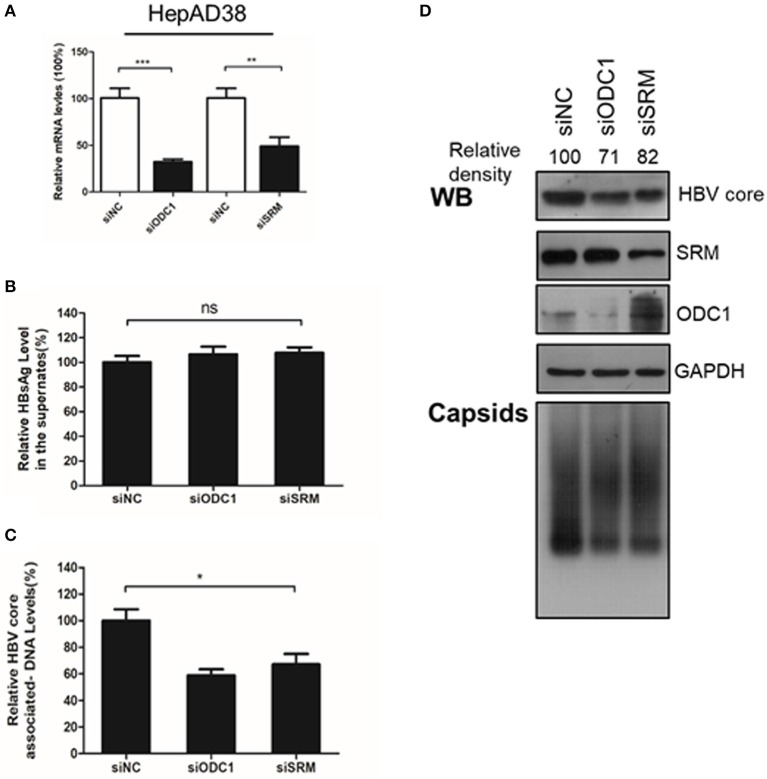
Silencing the expression of ODC1 and SRM restricted the HBV replication and reduced the HBc protein levels. **(A)** HepAD38 cells were transfected siRNA targeting ODC1 or SRM, and the knockdown efficiency was confirmed by using real-time RT-PCR. **(B)** The HBsAg levels in the supernatants were determined by ELISA methods. **(C)** The intracellular HBV DNA was extracted and measured by real-time PCR. **(D)** The protein levels of HBc, SRM and ODC1 were measured by Western blotting using relevant antibodies, as described in Figure 1. For viral capsid measurement, HBV particles were analyzed using a Native gel assay. Statistical significance was determined by one-way ANOVA with Tukey *post-hoc* test (*p < 0.05, **p < 0.01, ***p < 0.001; ns, not significant). Data have been represented as the mean ± SD of three independent experiments.

Next, we continued to test the potential function of ODC1 or SRM in regulating HBV replication. For that, the expression of ODC1 or SRM were knockdown by using specific siRNAs in HepAD38 (Figure 2A) and HBV markers, including HBsAg in the supernatant, the core-associated DNA levels or the HBc expression levels were then measured by ELISA, real-time PCR or Western blotting, respectively. As shown in Figures 2B–D, silencing of ODC1 or SRM in HepAD38 cells resulted in decreased viral DNA levels, as well as in the levels of the HBc protein and capsids, while it had no noticeable effect on the HBsAg levels in the supernatant. These findings suggest that ODC1 or SRM that regulating polyamine levels may be involved in HBV replication.

### DFMO Inhibits HBV Core-Associated DNA Replication by Reducing HBc Protein Levels

**Figure 3:**
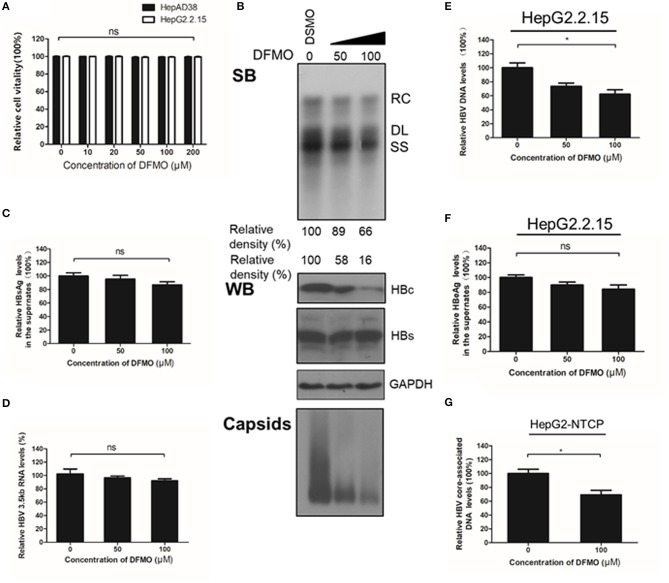
DFMO decreases the HBV core-associated DNA and the HBc protein levels. **(A)** Determination of cytotoxicity of HepAD38 and HepG2.2.15 cells treated DFMO measured by the MTS assay. **(B)** HepAD38 cells were treated with DFMO (50 μM, 100 μM), and the HBV core-associated DNA was extracted 3 days later and measured by Southern blot (upper panel). The levels of HBc and HBs were measured by Western blotting, and capsids levels were determined using a Native gel assay (lower panel) as described above. **(C)** The levels of HBsAg in supernatant were measured by ELISA assay as described above. **(D)** HBV 3.5kb RNA levels were measured by real-time RT-PCR. **(E)** and **(F)** HepG2.2.15 cells treated with DFMO (50, 100 μM) for 3 days, then the levels of intracellular HBV DNA or HBsAg levels in the supernatant were detected by real-time PCR **(E)** or ELISA assay **(F)**. **(G)** DFMO decreased the infection capacity of the HBV particles. HepAD38 cells in the absence of tetracycline were treated with DSMO or DFMO with indicated concentration for 3 days, and the HBV viral particles were then collected and added to the HepG2-NTCP cells. Five days later, the cytoplasmic viral DNA were extracted and measured by real-time PCR. RC, relaxed circular; DL, double stranded linear; SS, single stranded. Experiments were performed in triplicate, and data are represented as means ± SD. Statistical significance was determined by one-way ANOVA with the Tukey *post-hoc* test (*p < 0.05; ns, not significant).

To confirm the critical role of ODC1 in regulating HBV replication in hepatoma cells, we used DFMO, a specific inhibitor against ODC1. DFMO treatment had no impact on HBV-stably expressing cells HepAD38 or HepG2.2.15 cell viability even at high concentration of 200 μM (Figure 3A). The HepAD38 cells were firstly treated with DFMO under subtoxic concentration, and the HBV markers were then measured. As shown in Figure 3B, DFMO treatment reduced 34% of the viral DNA levels and 84% of the HBc protein levels at concentration of 100 μM. Similarly, the levels of viral capsids were also reduced significantly by DFMO treatment (Figure 3B). However, DFMO treatment had no effect on the levels of HBsAg in the supernatant or the HBV 3.5kb RNA levels (Figures 3C,D). Similar results were observed in HepG2.2.15 cells (Figures 3E,F). These results indicate that DFMO inhibits HBV core-associated DNA synthesis mostly by reducing HBc protein levels.

Next, to determine whether the decrease of HBc and HBV DNA levels had a potential impact on the infectiveness of this virus, HepAD38 cells were treated with DFMO for 3 days, followed by a collection of the HBV particles in the supernatants for an HepG2-NTCP cells infection. The cytoplasmic viral DNA was extracted and investigated by real-time PCR analysis. As shown in Figure 3G, the levels of viral DNA were decreased by 31% in the DFMO treatment group, suggesting that DFMO treatment reduced the infection capacity of viral particles. As DFMO target HBc, and nucleotide analogs such as LAM, and target HBV polymerase, we tested whether DFMO in combination with LAM led to the increased inhibition of the HBV DNA replication. Both LAM and DFMO reduced the HBV DNA levels. However, no significant combinatorial effect was observed (Supplement Figure 1).

### Polyamines Facilitate Viral Replication and HBc Protein Levels

**Figure 4:**
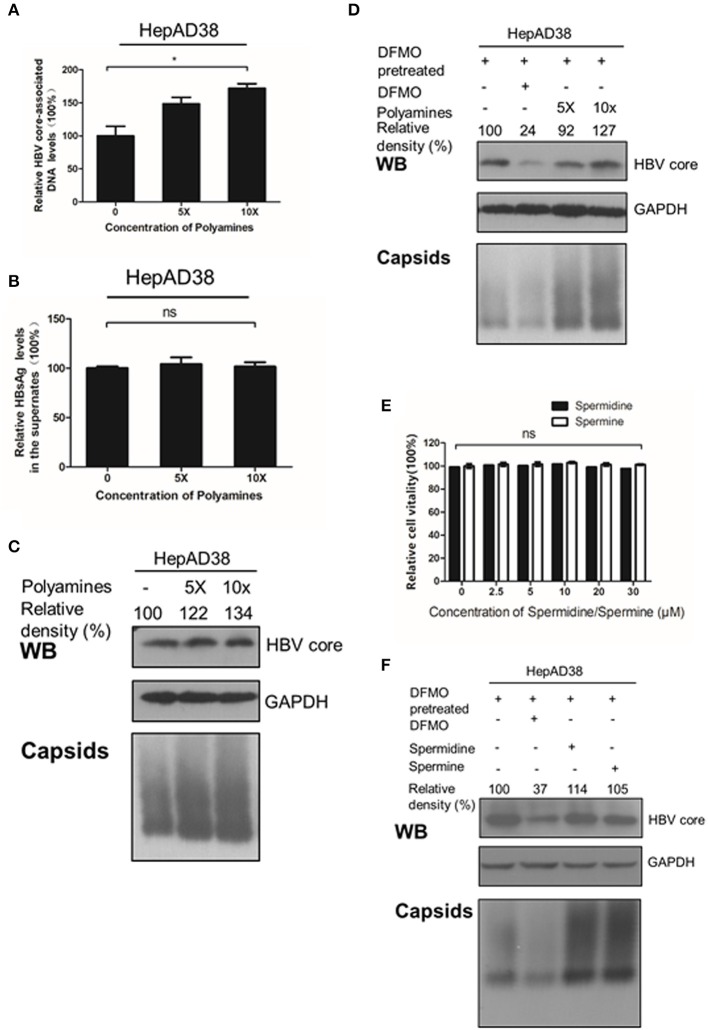
Polyamines enhances the HBc protein levels. **(A–C)** HepAD38 cells were treated with polyamines mixture for 3 days, then the levels of intracellular HBV DNA, HBsAg in supernatant, HBc or capsids were measured using real-time PCR **(A),(B)** ELISA assay or **(C)** Western blotting. **(D)** HepAD38 cells were pretreated with DFMO (100 μM) for 3 days, then divide in four groups: the control (lane 1), DFMO-treated group (lane 2), group replenished with a polyamine mixture (lane 3 and 4) for another 3 days, followed with Western blotting or Native gels to measure the levels of HBc or viral capsids as described above. **(E)** Determination of cytotoxicity of HepAD38 treated spermidine or spermine measured by using the MTS assay. **(F)** HepAD38 cells were pretreated for 3 days with DFMO (100 μM) to deplete the levels of polyamines, followed by an addition of DFMO (100 μM), exogenous spermidine (10 μM) or spermine (10 μM) for another 3 days, then the levels of HBc protein or capsids were determined as described above. Statistical significance was determined by one-way ANOVA with the Tukey *post-hoc* test (*p < 0.05, ns, not significant). Mean ± SD values from three independent experiments have been shown.

Considering that silencing of ODC1 or inhibition of polyamine biosynthesis by DFMO can deplete preexisting polyamines pools in cells (Gupta et al., 2016; Mounce et al., 2016b), we tested whether adding the exogenous polyamines could also affect HBV replication. First, HepAD38 cells were directly treated with polyamines for 3 days, and the intracellular viral DNA was extracted and measured by real-time PCR. As shown in Figures 4A–C, polyamines, without the DFMO pretreatment, had a minor effect of stimulating the viral DNA replication and the HBc protein levels, as well as moderate capsids levels, while no noticeable effect on HBsAg levels in the supernatant was observed. However, the addition of exogenous polyamines to the DFMO-pretreated HepAD38 cells significantly rescued the HBc protein and capsids levels (Figure 4D), suggesting HBV replication may requires threshold levels of polyamines.

Since spermine and spermidine are essential polyamines in mammals (Yuan et al., 2001; Moinard et al., 2005), then we investigated the potential function of spermine or spermidine toward an HBV replication. As expected, replenishment of spermidine or spermine at a subtoxic concentration to DFMO-pretreated HepAD38 cells rescued the HBc protein and capsids levels (Figures 4D,E), suggesting that polyamines stimulate viral replication by increasing HBc protein levels. Based on the data presented in Figures 2, 3, we conclude that polyamines were involved in HBV replication.

### DFMO Reduces of HBc Protein Levels Independent of Transcription and Translation Regulation

**Figure 5:**
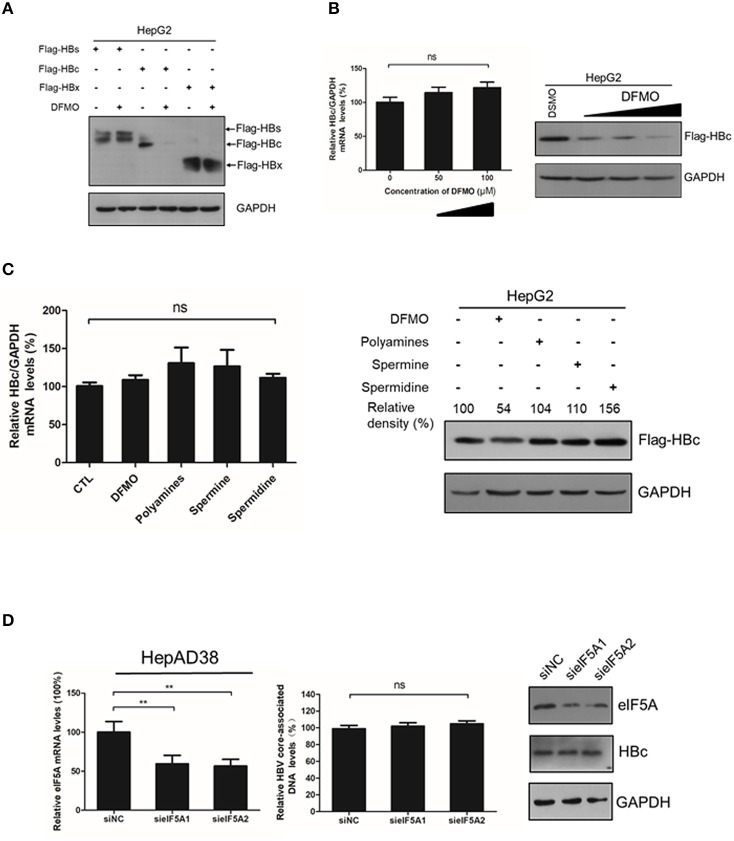
DFMO-mediated reduction of the HBc protein levels was independent of the transcription and translation pattern. **(A)** HepG2 cells were transfected with Flag-tagged HBs, Flag-tagged HBc, and Flag-tagged HBx plasmids, and treated with 50 μM DFMO for 3 days. Total proteins were extracted and subjected to Western Blotting. **(B)** HepG2 cells were transfected with Flag-tagged HBc plasmid, followed by DFMO treatment of different concentration for 3 days. The levels of HBc mRNA and protein were detected by using real-time RT-PCR (left panel) and Western blotting (right panel), respectively. **(C)** HepG2 cells were pretreated with 50 μM DFMO, then transfected with Flag-tagged HBc plasmid, followed by a replenishment using a polyamines mixture (5x), spermidine (10 μM) or spermine (10 μM) for another 3 days as described in Figure 4. The levels of HBc mRNA or proteins were determined by using real-time RT-PCR (left panel) or Western blotting (right panel). **(D)** HepAD38 cells were transfected siRNA targeting elF5A1 or elF5A2 and the levels of intracellular HBV DNA or HBc protein were determined by real-time PCR or Western blotting. Statistical significance was determined by using one-way ANOVA with the Tukey *post-hoc* test (*p < 0.05, ns, not significant). Mean ± SD values from three independent experiments have been shown.

As DFMO reduced, and polyamines increased the HBc protein levels Figures 3, 4, we investigated the underline mechanism of DFMO-mediated inhibition of the HBc protein levels. HepG2 cells were transfected with Flag-tagged HBc, HBx and HBs expression plasmids, and the cells were treated with 50 μM DFMO for 3 days. We observed that only HBc, but not for HBx or HBs protein levels were significantly reduced by DFMO treatment (Figure 5A), supporting our suggestion that DFMO inhibits viral replication mainly by targeting the HBc protein. In addition, DFMO treatment led to decreased protein levels of HBc without affecting its mRNA levels in HepG2 cells (Figure 5B), suggesting that DFMO inhibits HBc expression at the post-transcriptional level. Similar results were observed by replenishment spermidine or spermine to DFMO-pretreated HepG2 cells (Figure 5C).

It had been reported that spermidine is necessary for the hypusination of the eukaryotic initiation factor 5A(eIF5A) (Jao and Chen, 2006; Park, 2006). Hypusinated eIF5A is a translation factor that is currently regarded to be important for peptide chain elongation and broadly participates in the replication of multiple viruses, such as HIV, EBOV and HSV-1 (Malim et al., 1990; Olsen et al., 2016; Mounce et al., 2017). In mammalian cells, there are two isoforms of eIF5A, eIF5A1 and eIF5A2, which share 84% homology and both harbor the hypusine modification (Caraglia et al., 2013). We examined whether eIF5A was necessary for the HBc protein translation. However, silencing the expression of eIF5A1 or eIF5A2 had no noticeable effect on HBc protein levels or viral DNA replication (Figure 5D), which indicated that DFMO inhibited HBc protein levels independent of hypusination modification of eIF5A by spermidine.

### DFMO Treatment Decreases the HBc Protein Stability

**Figure 6:**
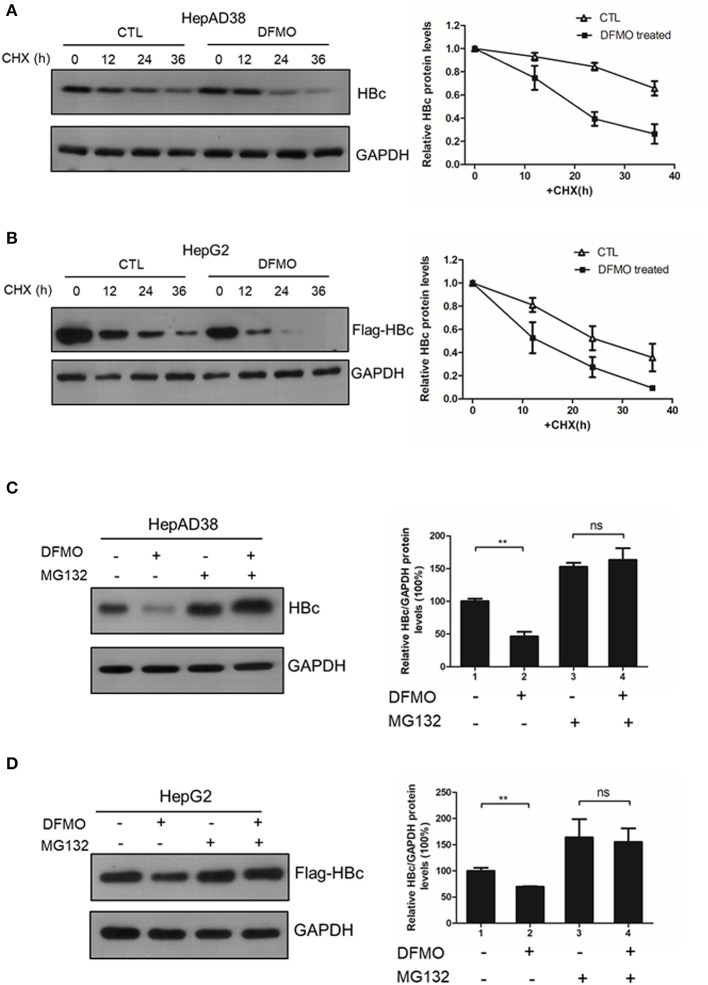
DFMO decreased the HBc protein stability. **(A)** HepAD38 cells were treated with 100 μM DFMO in the absence of tetracycline for 3 days, and cultured then with 100 μg/mL CHX for indicated time periods. The steady-state levels of HBc were determined by Western blotting (left panel). Quantification of the HBc protein by ImageJ has been shown in the right panel. **(B)** HepG2 cells were treated with 50 μM DFMO for 3 days, then transfected with Flag-tagged HBc, followed with 100 μg/mL CHX treatment as described in A. **(C)** HepAD38 cells were treated with 100 μM DFMO for 3 days in the absence of tetracycline for 3 days, followed by a treatment with 10 μM MG132. The levels of HBc were measured by Western blotting analysis. **(D)** HepG2 cells treated with 50 μM DFMO, transfected with Flag-tagged HBc, cultured with MG132 as described in **(C)**. The levels of HBc were measured by Western blotting analysis. Statistical significance was determined by one-way ANOVA with the Tukey *post-hoc* test (*p < 0.05, ns, not significant). Mean ± SD values from three independent experiments have been shown.

Finally, we investigated whether DFMO treatment affected the HBc protein stability. HepAD38 cells were pretreated with DFMO for 3 days, and after the removal of tetracycline from the medium to initiate HBV replication, cycloheximide (CHX) was added to the cells in order to block protein synthesis. As shown in Figure 6A, DFMO treatment significantly reduced the half-life of HBc protein in HepAD38 cells. Similar results were obtained in the HepG2 cells transfected with Flag-tagged HBc expression plasmid (Figure 6B). These findings suggested that DFMO inhibited the HBc protein levels by reducing its stability.

Next, we used a proteasome inhibitor MG132 to determine whether DFMO-mediated HBc degradation was dependent on the proteasome pathway. Indeed, the effects of DFMO treatment on downregulating the levels of HBc protein were abolished by MG132 treatment both in the HepAD38 and HepG2 cells (Figures 6C,D), indicating that DFMO inhibited the HBc protein levels via promoting its ubiquitination.

## Discussion

It is known that viruses can utilize host cell resources for their own replication. Understanding this process in more detail can provide strategies for the development of antiviral treatment. Polyamines play various roles within the mammalian cells, including gene transcription and mRNA translation (Childs et al., 2003; Pegg, 2009; Igarashi and Kashiwagi, 2015; Kashiwagi et al., 2018). The intracellular levels of polyamines are regulated by several rate-limiting enzymes such as ODC1, SRM and SMS. Recent reports have indicated that viral infection can affect polyamine synthesis, for instance via influencing the expression of the enzymes involved in regulation of the polyamine biosynthetic pathway and polyamine levels, and, as a consequence, contribute to their own replication during viral life cycle (Mounce et al., 2017). For example, the levels of ODC1 have been reported to increase in adenovirus-infected cells (Liu et al., 1985). Similarly, HCMV infection also stimulates the activity of ODC1 (Isom, 1979) and an increment of the spermine and spermidine levels (Clarke and Tyms, 1991). In contrast, the expression of ODC1, as well as the levels of spermine and spermidine have been reported to be reduced in cells harboring a full-length HCV replicon (Smirnova et al., 2017). In our study, we found that the expression of ODC1 and SRM were both upregulated in the HBV replication cells and cell models of infection (Figure 1). Furthermore, silencing the expression of ODC1 or SRM (Figure 2), as well as inhibition of polyamine biosynthesis with DFMO treatment (Figure 3), affected the replication of the viral intracellular DNA, the levels of the HBc protein and capsids, suggesting that HBV infection could utilize polyamine synthesis to regulate its replication. For the further mechanistic clarification of the polyamine metabolism, the cells pretreated with DFMO were replenished with exogenous polyamines, including the spermine or spermidine, which significantly rescued the HBc protein and capsids levels (Figure 4). This observation strengthens the important role of polyamines in promoting HBV replication during the HBV life cycle. However, supplementing cells directly with polyamines had slight effect on the replication of the viral DNA and the HBc protein levels and moderate influence on the capsids levels (Figure 4), suggesting that HBV replication required a threshold levels of polyamines. Similar results were observed for polyamines in CHIKV replication (Mounce et al., 2016b).

As a new emerging anti-viral drugs, DFMO inhibits replication of several viruses via complex pathways: for DNA virus, such as HCMV, DFMO represses the production of viruses by interfering with the viral assembly (Tyms and Williamson, 1982; Gibson et al., 1984); while for RNA viruses, such as CHIKV and ZIKV, DFMO exhibits broad spectrum of antiviral functions by depleting the polyamines pools (Mounce et al., 2016a). In addition, hypusination of eIF5A, a unique posttranslational modification of an aminobutyl moiety from the spermidine at the Lys50 site via deoxyhypusine synthase (DHPS), is necessary for viral protein translation (Olsen and Connor, 2017). As a result, reducing the levels of spermidine by DFMO treatment can also inhibits the expression of EBOV minigenome (Olsen et al., 2016, 2018). In our research, we revealed that DFMO reduces the HBc protein levels by promoting its ubiquitination (Figure 6), as a consequence, inhibiting HBV capsids levels and DNA replication (Figure 3). This finding provides a new insight into DFMO function against viral replication. It has been reported that HBc protein can be modified by ubiquitination, and lysine K7 and K96 were potential target sites for ubiquitination (Rost et al., 2006; Lubyova et al., 2017). In addition, Np95/ICBP90-like RING finger protein (NIRF), a novel E3 ubiquitin ligase, which could promote HBc protein degradation via binding to HBc (Qian et al., 2012). In our study, we found DFMO inhibited the HBc protein levels via promoting its ubiquitination. As DFMO is an inhibitor of ODC1, which would affect the cellular polyamines levels, it is possible that ODC1/polyamines may regulating the ubiquitination of HBc by affect the expression of E3 ubiquitin ligase such as NIRF. Further work for identification of DFMO role in the ubiquitination of HBc protein will help to clarify this mechanism. Of note, the concentration of DFMO for inhibition of HBV replication applied in our study was relatively high (100 μM), meanwhile, the concentration of 500 μM was used in other studies for testing a DFMO-mediated viral RNA replication (Mounce et al., 2016a). As the human ODC1 has a rapid turnover (t1/2 <1 h) (Heby et al., 2003), it is necessary to apply high doses of the treatment in these studies.

In summary, our findings demonstrate that DFMO restricts the HBV replication via targeting the HBc stability, which highlights the importance of DFMO and reveal a new mechanism against viral replication. Given that DFMO is the Food and Drug Administration (FDA)-approved drug used to treat female facial hirsutism (Wolf et al., 2007), human African trypanosomiasis (Pepin et al., 1987), and some cancers (Casero and Woster, 2009). More importantly, DFMO is safe to use and is well tolerated in humans (Creaven et al., 1993), which highlights the its potential values as an anti-HBV therapy.

## Data Availability Statement

All datasets generated for this study are included in the article/Supplementary Material.

## Author Contributions

BM and YH conceived the project. BM, ZW and YH designed the study and analyzed the data. BM, ZW, SP, QL and KC performed the study. JC, AH and YH provided the funds. BM prepared the draft manuscript. YH edited and revised the manuscript.

### Conflict of Interest

The authors declare that the research was conducted in the absence of any commercial or financial relationships that could be construed as a potential conflict of interest.

## Supplementary Material

Figure S1 DFMO does not show combinational effect of inhibiting HBV DNA replication with LAM. HepAD38 cells were treated with DFMO (100 μM) or/and LAM (0.5 μM) for 3 days, and HBV core-associated DNA was extracted and measured by real-time PCR.
