# Supplementary figures and images for: Difluoromethylornithine, a Decarboxylase 1 Inhibitor, Suppresses Hepatitis B Virus Replication by Reducing HBc Protein Levels
Source: Front Cell Infect Microbiol. 2020 Apr 16;10:158. doi: 10.3389/fcimb.2020.00158 (PMC7176913; doi:10.3389/fcimb.2020.00158)

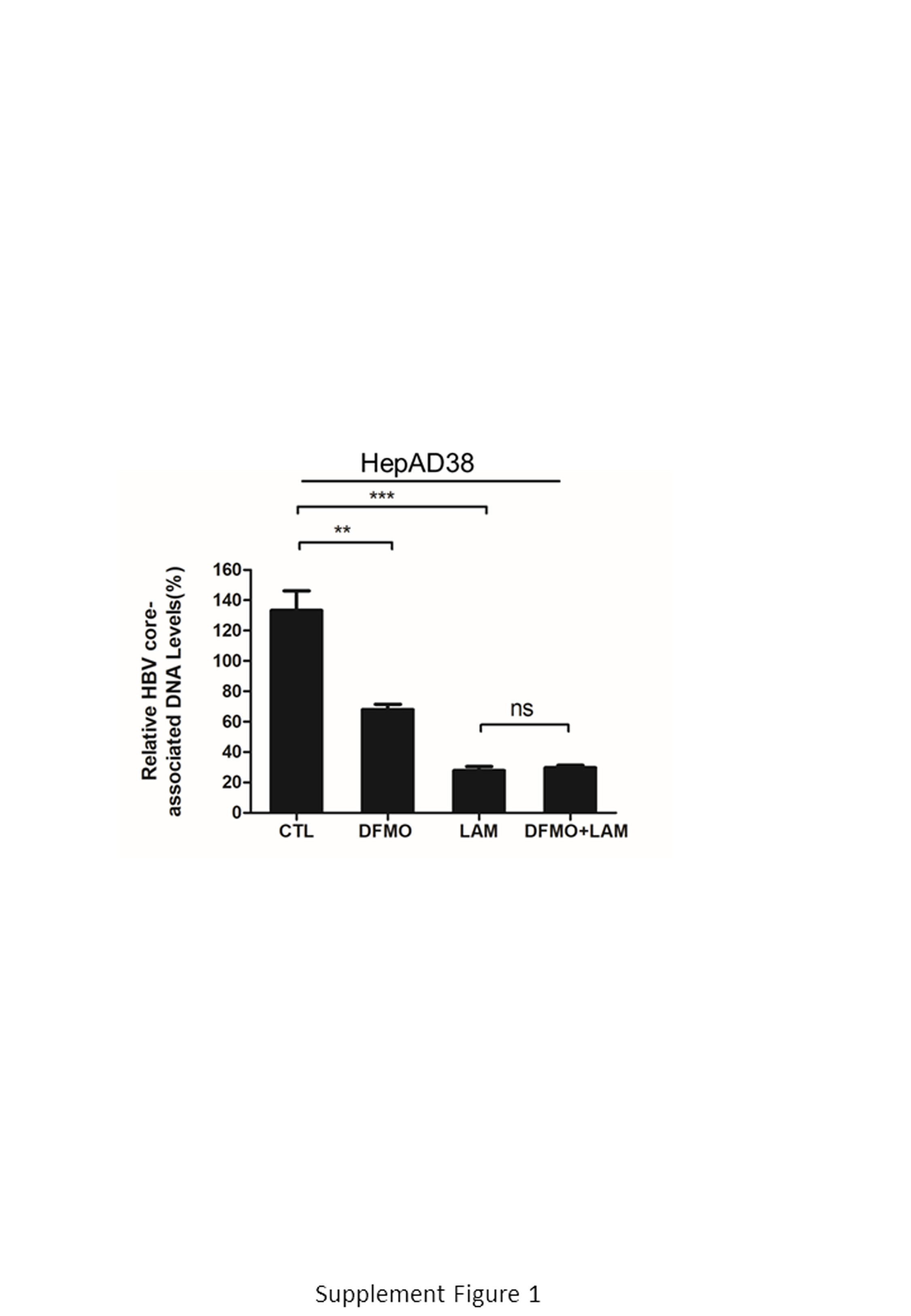

Supplement: Figure S1 — DFMO does not show combinational effect of inhibiting HBV DNA replication with LAM. HepAD38 cells were treated with DFMO (100 μM) or/and LAM (0.5 μM) for 3 days, and HBV core-associated DNA was extracted and measured by real-time PCR. [file Image_1.TIF]
