# Supplementary material for: Difluoromethylornithine, a Decarboxylase 1 Inhibitor, Suppresses Hepatitis B Virus Replication by Reducing HBc Protein Levels
Source: Front Cell Infect Microbiol. 2020 Apr 16;10:158. doi: 10.3389/fcimb.2020.00158 (PMC7176913; doi:10.3389/fcimb.2020.00158)
Supplement: Supplementary file 2 [file Table_1.doc]

**Supplement Table 1. SiRNA sequence of target gene and real-time PCR primer sequence**

| **Name** | **Sequence** |
| --- | --- |
| **si-ODC1** | sense:5’- GCUGUGACCUGCCUGAAAU-3’ |
|  | anti-sense:5’- AUUUCAGGCAGGUCACAGC-3’ |
| **si-SRM** | sense:5’-GGAGUCCUAUUACCAGCUC -3’ |
|  | anti-sense:5’-GAGCUGGUCCUAGGACUCC -3’ |
| **si-** **elF5A1** | sense:5’- AAAGGAAUGACUUCCAGCUGA-3’ |
|  | anti-sense:5’-UCAGCUGGAAGUCAUUCCUUU -3’ |
| **si-** **elF5A2** | sense:5’-GCUUCCAGCACUUACCCUA -3’ |
|  | anti-sense:5’-UCGGGUAAGUGCUGGAAGC -3’ |
| **ODC1** | sense:5’-TGTTGCTGCTGCCTCTACGTT-3’ |
|  | anti-sense:5’-GCTGGCATCCTGTTCCTCTACTT-3’ |
| **SRM** | sense:5’-CCCTCCGTGGAGTCCGTGGTC -3’ |
|  | anti-sense:5’-CTGGCAGGAACTTCTTGGAGACTTG-3’ |
| **SMS** | sense:5’- TGGAAATATTCTCATCCTTAGTGGG-3’ |
|  | anti-sense:5’-CGGGTATATGCCAAATCACTCTCT -3’ |
| **elF5A1** | sense:5’-ACGTTTGGAATCGAAGCCTCT -3’ |
|  | anti-sense:5’-CCTTTGAGCACCACAAAGCC -3’ |
| **elF5A2** | sense:5’-GCTTCGTGGTGCTCAAAGGA -3’ |
|  | anti-sense:5’- TGCCTAGTTCACCTTCTGGC-3’ |
| **HBc** | sense:5’-CCTAGTAGTCAGTTATGTCAAC -3’ |
|  | anti-sense:5’-TCTATAAGCTGGAGGAGTGCGA -3’ |
| **GAPDH** | sense:5’-GAAGGTGAAGGTCGGAGTC -3’ |
|  | anti-sense:5’- GAAGATGGTGATGGGATTTC-3’ |
| **HBV DNA** | sense:5’-TGCGGCGTTTTATCATATTCC -3’ |
|  | anti-sense:5’-ATACCTTGGTAGTC CAGAAGAACCA -3’ |
